# Diagnosis and treatment of transthyretin‐related amyloidosis cardiomyopathy

**DOI:** 10.1002/clc.23434

**Published:** 2020-07-29

**Authors:** Catherine Teng, Pengyang Li, Ju Young Bae, Su Pan, Richard A. F. Dixon, Qi Liu

**Affiliations:** ^1^ Department of Medicine Yale New Haven Health‐Greenwich Hospital Greenwich Connecticut USA; ^2^ Department of Medicine Saint Vincent Hospital Worcester Massachusetts USA; ^3^ Molecular Cardiology Research Texas Heart Institute Houston Texas USA

**Keywords:** heart disease, physical diagnosis/cardiovascular, treatment

## Abstract

Transthyretin‐related amyloidosis (ATTR) is a subgroup of amyloidosis that results from extracellular misassembled and toxic amyloid deposits affecting multiple organ systems, and cardiac tissues in particular. Because ATTR often presents as heart failure with preserved ejection fraction (HFpEF), it has been largely underdiagnosed. Once considered incurable with a grave prognosis, ATTR cardiomyopathy has seen the development of promising alternatives for diagnosis and treatment, with early diagnosis and treatment of ATTR cardiomyopathy highly beneficial due to its high mortality rate. For instance, diagnosing ATTR cardiomyopathy previously required a cardiac biopsy, but new modalities, such as cardiac magnetic resonance imaging and radionuclide bone scans, show promise in accurately diagnosing ATTR cardiomyopathy. Ongoing research and clinical trials have focused on identifying new treatments which primarily target amyloid fiber formation by inhibiting TTR gene expression, stabilizing the TTR tetramer, preventing oligomer aggregation, or affecting degradation of amyloid fibers. In this review, we describe the advances made in the diagnosis and treatment of ATTR in order to increase awareness of the disease and encourage a lower threshold for ATTR workup. Our review also highlights the need for improving the screening, diagnosis, and treatment guidelines for ATTR cardiomyopathy.

## 1 BACKGROUND

Transthyretin (TTR)‐related amyloidosis (ATTR), a rare and underdiagnosed disease, mainly affects the cardiac and peripheral nerves and is fatal if not treated in time.^1^ Primarily synthesized in the liver, TTR is a protein that transports vitamin A and thyroxine in plasma and cerebrospinal fluid.^2^ The mechanism of transportation involves four oligomers that attach to each other to form two dimers, which in turn form a TTR tetramer that contains the hormone binding site.^2^ Through gene mutation or aging of the protein, TTR can become unstable and break up into oligomers, which aggregate into insoluble fibrils called TTR amyloid that deposit into extracellular space or tissues, causing ATTR.^3^

Based on the type of precursor and misfolded protein amyloids, there are different types of amyloid diseases from ATTR to light chain amyloidosis (AL), which is caused by aggregation of immunoglobulin light chains produced by plasma cells in the bone marrow.^4^ Depending on the type of precursor protein, different protein amyloids target different organ systems, although the exact mechanisms involved in the targeting are not completely understood. ATTR almost exclusively infiltrates cardiac tissue, peripheral nerves, and autonomic nerves but can occasionally deposit in the digestive system and kidneys.^4^

ATTR and AL are the most common causes of cardiac amyloidosis.^5^ When ATTR infiltrates cardiac tissues, the heart stiffens, causing heart failure with preserved ejection fraction (HFpEF). In addition, cardiomyopathy can result from the direct toxicity of the prefibrillary proteins in ATTR, which interfere with the contractility and relaxation of the heart.^6^

Diagnosing ATTR cardiomyopathy can be challenging because its presentation can resemble more common cardiac‐related diagnoses, especially congestive heart failure.^7^ Because ATTR cardiomyopathy has a grave prognosis with most deaths caused by cardiac‐related issues, recognition and referral to a cardiologist for the management are important as early suspicion of ATTR cardiomyopathy could be lifesaving. Moreover, over the last few years, novel treatment measures and robust scientific and clinical research now allow for a lower threshold for workup if the initial diagnosis is unclear.

## 2 ATTR SUBGROUPS

ATTR can be divided into two groups: wild‐type ATTR (ATTR‐wt) and mutated ATTR.^5^ ATTR‐wt is most commonly seen in elderly men over age of 70.^8^ The mechanism of ATTR‐wt involves aging‐related protein misfolding and ATTR deposition. The cause of the change in proteins during the aging process is unknown, but this type of ATTR is generally not considered hereditary. Compared to mutated ATTR, ATTR‐wt is characterized by a greater left ventricular wall thickness, a more substantial reduction in ejection fraction, and a higher degree of longitudinal strain.^9^ Although ATTR deposits can be found throughout the body, cardiac deposits are the most common.^10^ Patients with ATTR‐wt may also have musculoskeletal issues including carpal tunnel syndrome and lumbar spinal stenosis.^10^ In addition, patients can present with gastroenterological symptoms, but to a much lesser extent than seen in mutated ATTR.^11^

In contrast, the inheritance of the TTR mutation gives rise to mutated ATTR‐ararer condition and the primary cause of familial amyloid cardiomyopathy.^12^ Unlike in ATTR‐wt, the age of onset in this condition typically varies from 30 to 80 years old depending on the type of mutation.^10^ The gene for TTR is located on chromosome 18 (18q12.1), and its more than 100 variable mutations show an autosomal dominant pattern.^3^ The wide spectrum of mutations varies with geographic region in mutated ATTR, which has great genotypic and phenotypic heterogeneity.^13^ The most common mutation in the United States is the ATTR V122I variant, which is seen in 3% to 4% of African Americans.^14^ This type of mutated ATTR is often associated with cardiomyopathy.^14^ Another common variant, ATTR V30M, is often found in Japan, Portugal, Spain, and descendants of those regions. It typically causes peripheral neuropathy‐related symptoms.^14^ The ATTR T60A variant is the most frequent in the United Kingdom^3^ and the second most common in the United States.^15^ This type of mutation often affects cardiac, peripheral nervous, and autonomic nervous systems and corresponds to an especially high rate of carpal tunnel syndrome.^3^ ATTR‐wt and mutated ATTR are differentiated by gene sequencing.

## 3 EPIDEMIOLOGY

The true prevalence of ATTR cardiomyopathy is unknown because it is largely underdiagnosed.

As much as 10% to 20% of the population over age 65 may be affected by ATTR‐wt, which leads to ATTR cardiomyopathy manifested as congestive heart failure.^16^ Evidence suggests that ATTR‐wt could be the most common type of amyloid‐related cardiomyopathy and is especially prevalent among patients with HFpEF.^17^

In a study conducted in 2014 by Mohammed et al, the incidence of ATTR‐wt increased with age, and 17% of patients with HFpEF also had TTR amyloid deposits in the myocardium.^17^ In another study, 13% of elderly adults with HFpEF had ATTR cardiomyopathy as shown by bone scintigraphy.^18^

## 4 CLINICAL MANIFESTATIONS

Clinical manifestations of ATTR are nonspecific and vague. Patients can present with only cardiac involvement, only peripheral or autonomic neuropathy, or a combination based on the type of ATTR and mutation.^8^

Patients with ATTR cardiomyopathy present with classic signs of heart failure, including shortness of breath, orthopnea, fatigue, and peripheral edema. In addition to symptoms of HFpEF, ATTR can be accompanied by atrial fibrillation, syncope, conduction system disease, and stroke; these are thought to result from the infiltration of ATTR into cardiac tissue and the peripheral nervous system. Peripheral neuropathy such as numbness, paresthesia, and pain, and autonomic dysfunction such as orthostatic hypotension, and bowel/bladder dysfunction are also seen in these patients.^19^

## 5 DIAGNOSTIC APPROACH

The diagnosis of ATTR cardiomyopathy is challenging because patients present with a constellation of symptoms seen in more common conditions, such as hypertensive left ventricular hypertrophy, carpal tunnel syndrome, or idiopathic HFpEF. Clinicians are trained to attribute these nonspecific manifestations to more common diagnoses. In addition, the lack of consensus in screening criteria poses another barrier to routinely considering this condition in a diagnostic workup.

Clinicians have now recognized that a high index of suspicion for recognizing the disease is required to diagnose ATTR cardiomyopathy.^20^ Maurer et al suggested that ATTR cardiomyopathy should be suspected in all patients with heart failure who have unexplained increased left ventricular wall thickness and a nondilated left ventricle.^20^ In addition, even without a typical restrictive pattern seen on noninvasive modalities, patients may still have underlying ATTR cardiomyopathy manifesting as heart failure and/or arrhythmia.

### 5.1 Tissue biopsy

A diagnosis of ATTR cardiomyopathy has previously required a cardiac biopsy. Given the perceived rarity of the disease and the low positive predictive value of this invasive diagnostic approach, cardiac biopsy has not been frequently used, which has perhaps contributed to the underdiagnosis of ATTR. When the decision is made to perform a biopsy, the most minimally invasive approach is often used. Common biopsy sites are the salivary gland, the subcutaneous fatty tissue of the abdominal wall, or the rectal, kidney, or gastric mucosa.^21^ Tissue biopsy has a relatively lower sensitivity for ATTR than for AL.^22^ Newer, more noninvasive alternatives are currently available to confirm the diagnosis of ATTR amyloidosis, as discussed below.

### 5.2 Echocardiogram

Echocardiography remains an excellent screening tool for ATTR cardiomyopathy given its cost‐effectiveness, accessibility, and lack of radiation. It is the initial diagnostic test of choice when cardiac amyloidosis is suspected.^23^ Previously viewed as a modality with limited sensitivity and diagnostic specificity, echocardiography now has new parameters designed to provide a more sensitive measurement of left ventricular function.

Early findings on echocardiogram usually include a nondilated left ventricle with a concentrically thickened myocardium characterized by increased echogenicity, thickening of the right ventricular free wall, and visibly dilated atria and interatrial septum.^4^ In addition, parameters have been identified for the diagnosis of cardiac amyloidosis using two‐dimensional speckle‐tracking echocardiography. According to Pagourelias et al, the ejection fraction global longitudinal strain (LS) ratio has the best accuracy to detect cardiac amyloidosis in patients with ventricular hypertrophy; it has an estimated sensitivity of 89.7% and specificity of 91.7%.^24^ Apical sparing, a pattern characterized as LS reduction of the basal wall segment sparing apical segments, is calculated as the ratio between apical LS and basal and mid‐ventricle LS.^25^, ^26^ A ratio of 1 is thought to show high sensitivity (93%) and specificity (82%) for the diagnosis of cardiac amyloidosis^27^ and independently predicts major adverse cardiac events.^28^ Apical LS abnormalities are similar across different amyloidosis types and reflect the amyloid deposition burden.^28^

### 5.3 Cardiac magnetic resonance imaging

Cardiac magnetic resonance imaging (MRI) is a sensitive modality in diagnosing cardiac amyloidosis. The key features of amyloidosis cardiomyopathy on cardiac MRI are increased left ventricular wall thickness, transmural or subendocardial late gadolinium enhancement, and elevated native T1 and extracellular volume fraction.^29^ Studies have shown that cardiac MRI has a 86% to 88% sensitivity and a 86% to 90% specificity for this diagnosis^30^; however, this modality is not widely available at this time. In addition, although cardiac MRI can clearly show late transmural gadolinium enhancement,^31^ it does not differentiate between the types of amyloidosis,^32^ and the amyloid deposit burden is somewhat underestimated compared to radionuclide bone scan.^33^

### 5.4 Radionuclide bone scan

Some data suggests that cardiac uptake on a radionuclide bone scan is >99% sensitive but not specific for ATTR cardiomyopathy.^34^ The specificity of cardiac uptake grade 2 or 3 was estimated to be approximately 87%.^34^ In another study of 99mTc‐pyrophosphate (^99m^Tc‐PYP) as a cardiac imaging tracer, the sensitivity was 97%, with 100% specificity.^35^ Radionuclide bone scan with 99mTc‐labeled bisphosphonate (^99m^Tc‐MDP) was noted to localize ATTR deposits by the Perugini grading system, as ^99m^Tc‐MDP preferentially binds to ATTR.^36^, ^37^ In addition to 100% sensitivity and specificity, the tracer technetium‐99 m‐labeled 3,3‐diphosphono‐1,2‐propanodicarboxylic acid (^99m^Tc‐DPD) was more accurate in identifying the amyloid deposit burden than was MRI. In the United States, given that ^99m^Tc‐MDP is widely available in routine bone scans, some experts argue that it would be a more feasible option than cardiac MRI in clinical practice.^32^ Furthermore, preliminary data suggests that grade 2 or 3 cardiac uptake on radionuclide bone scan in the absence of monoclonal protein as shown on serum tests and urine immunofixation is diagnostic of ATTR cardiomyopathy.^34^

## 6 TREATMENT

Once considered untreatable, ATTR amyloidosis now can be managed with contemporary therapies that improve survival rate and halt disease progression. Currently, multiple drugs are in various stages of clinical trials, and these new treatments may improve the prognosis of patients with a confirmed diagnosis of ATTR amyloidosis (Table 1).

**TABLE 1:** Drug therapies for ATTR

Drug	Dosage	Mechanism of action	Potential adverse effects	Stage of clinical trial/FDA approval	Reference
Inhibitors of TTR gene expression					
Patisiran	∼80‐min IV infusion based on actual body weight: <100 kg; 0.3 mg/kg ≥100 kg; 30 mg	siRNA inhibiting the expression of both variant and wt‐TTR	URTI, AV block, infusion‐related reactions, erythema, muscle spasm, keratoconjunctivitis sicca, vitamin A deficiency	FDA approved	Adams et al 2018
Inotersen	284‐mg subcutaneous injection once weekly	Antisense oligonucleotides causing degradation of mutant and wt‐TTR mRNA	Peripheral edema, arrythmia, presyncope, headache, nausea, vomiting, thrombocytopenia, glomerulonephritis, dyspnea	FDA approved for neuropathy; phase 2 trial ongoing for ATTR cardiomyopathy	Benson et al 2017
Revusiran	500‐mg subcutaneous injection	siRNA inhibiting the expression of both variant and wt‐TTR	Nausea, vomiting, constipation, injection site pain	Phase 3 trial (Discontinued)	Sutherland 2020
Tetramer stabilizers					
Tafamidis	VYNDAQEL 80 mg (four 20‐mg tafamidis meglumine capsules) orally once daily or VYNDAMAX 61 mg (61‐mg tafamidis capsule) orally once daily	Blocks the rate‐limiting step in TTR genesis by inhibition of TTR tetramer dissociation	Hypothyroidism, skin ulcer	FDA approved	Emdin et al 2019
Diflunisal	Diflusinal 250 mg two times daily + histamine receptor antagonist or a proton pump inhibitor	Nonselective binding to T4 site of TTR for preventing amyloid fibril formation	Headache, dizziness, skin rash, diarrhea, tinnitus	Phase 2, phase 3 (Completed)	Berk et al 2017
AG10	(0.1, 0.3, 1, 3, 10, and 30 mg/kg) IV every 28 d	Monoclonal antibody specifically targeting TTR amyloid deposit	No results posted	Phase 1, open label study (recruiting)	Fox et al 2020
Inhibitors of oligomer aggregation					
Epigallocatechin gallate (EGCG)	Drinking 1.5‐2 L of green tea daily OR intake of capsules containing 300 mg GTE	Binds to soluble TTR and decreases oligomer aggregation into amyloid fibers	Hypoglycemia, liver and renal failure, hypochromic anemia	Observational study; no clinical trial registered to date	ClinicalTrials.gov 2020
Inhibitors of degradation and reabsorption of amyloid fibers					
Doxycycline‐tauroursodeoxycholic acid (Doxy/TUDCA)	Doxycycline (100 mg twice daily) Taurousodeoxycholic acid (250 mg three times daily)	Complete disaggregation of amyloid fibers with generation of nontoxic molecular species	No results posted	Phase 3	Wixner et al 2019
Miridesap	200 mg oral once daily	Targeting serum amyloid P protein or amyloid fibrils by an antibody	No results posted	Phase 2 (terminated)	Emdin et al 2019
PRX004	Starting dose of 0.1 mg/kg, once every 28 d for total of 3 doses	Monoclonal antibody targeting TTR amyloid deposits inhibiting TTR fibrillogenesis	No results posted	Phase 1 open label study (recruiting)	ClinialTrials.gov, 2019
Dezamizumab	20 mg/h IV infusion for up to 72 h, followed by 60 mg three times daily subcutaneously for 8 d	Humanized monoclonal IgG1 antiserum amyloid P component, which activates and triggers clearance of amyloid	Headache, restless leg syndrome, diarrhea, dry eye, urticarial vasculitis, dactylitis, injection site bruising	Phase 2 (terminated)	ClinicalTrials.gov 2019

Abbreviations: ATTR, transthyretin‐related amyloidosis; IV, intravenous; TTR, transthyretin; URTI, upper respiratory tract infection; wt, wild‐type.

### 6.1 Medical treatment of ATTR

Most medications being developed or tested in ongoing clinical trials have focused on either preventing the formation of the TTR‐related amyloid protein or inhibiting the degradation of the TTR tetramer. The tetramer is the four oligomer unit that normally forms the binding site for thyroxine and vitamin A in plasma and cerebrospinal fluid. The mutation or aging of TTR can result in instability of the tetramer, which, in turn, may cause the tetramer to breakdown and form an insoluble beta‐pleated sheet, which frequently infiltrates the myocardium.^38^

#### 6.1.1 Drugs that inhibit TTR gene expression

Patisiran and revusiran are small interfering ribonucleic acids (siRNAs) that work to block the expression of both variant and wild‐type TTR.^39^ Revusiran was discontinued after an imbalance in death between the two treatment arms was discovered in the ENDEAVOR phase 3 clinical trial.^40^ In contrast, inotersen is an antisense oligonucleotide that binds to the mRNA from which TTR is normally synthesized.^41^ Inotersen appears to be effective in inhibiting both variant and wild‐type TTR protein production. In phase 3 trials, inotersen received FDA approval for treating patients with ATTR‐related polyneuropathy; however, the study was not sufficiently powered to allow for assessing the effect of the drug on cardiomyopathy.^41^ The tolerability and efficacy of inotersen for ATTR cardiomyopathy are now being tested in a 24‐month open label phase 2 clinical trial at Brigham and Women's Hospital (NCT03702829).^42^

#### 6.1.2 Drugs that stabilize the tetramer

##### Tafamidis

Tafamidis, a selective tetramer stabilizer, is a small molecule that blocks the rate‐limiting step in TTR genesis by binding to the T4 binding sites to inhibit the dissociation of TTR tetramers.^43^ In May 2019, tafamidis was officially approved by the FDA for treating cardiomyopathy caused by either wild‐type or hereditary ATTR.^43^ In a clinical trial, after 30 months, patients receiving tafamidis showed a 13.4% absolute reduction in overall mortality and a 22% reduction in the yearly cardiovascular hospitalization rate.^44^

Evidence suggests that tafamidis treatment may be more effective in patients with New York Heart Association (NYHA) class I/II heart failure than in those with class III disease.^44^ The efficacy and safety in patients with NYHA class IV heart failure has not been established.^44^ Therefore, early suspicion for ATTR is paramount for making a timely diagnosis and initiating early treatment.

Tafamidis is currently under postmarketing surveillance. As it is a new medication, most physicians have yet to familiarize themselves with its general indications, doses, and side effects. In Japan, as a general rule, only institutions with a multidisciplinary team that performs more than 15 myocardial biopsies annually in suspected cases are permitted to start administering tafamidis.^44^

##### Diflunisal

Diflunisal, a nonsteroidal anti‐inflammatory drug (NSAID), has been shown to nonselectively bind to the T4 binding site of TTR and stabilize TTR tetramers to prevent amyloid fibril formation in vitro.^45^ Given the nature of NSAIDs, the use of diflunisal has been controversial due to its inhibition of cyclooxygenase enzymes and its related gastrointestinal bleeding and renal dysfunction.^45^ In a phase 2, small open label prospective study at Columbia University, 77 patients were treated with diflusinal (250 mg twice daily) along with either a histamine receptor antagonist or a proton pump inhibitor between June 2009 and December 2011.^45^ After a 3‐month follow‐up, diflunisal‐treated patients had no hospitalizations for worsening heart failure, no significant changes in cardiac function, and a slight increase in brain natriuretic peptide and troponin I.^45^ Diflunisal was well tolerated, and patients showed no significant change in renal function or bleeding risk as measured by the estimated glomerular filtration rate (eGFR) and hemoglobin, respectively.^46^ In a clinical trial in France, diflunisal reduced the neurologic impairment aspect of patients with ATTR.^47^, ^48^, ^49^ Phase 2 and phase 3 studies have been conducted by Berk et al, but diflunisal has not yet received FDA approval for use in ATTR.^50^

##### AG10

AG10 is a potent selective kinetic stabilizer of TTR.^16^ In in vitro studies, Penchala et al showed that AG10 prevented the disassociation of both wild‐type TTR and V122I mutation‐related TTR.^16^ In a randomized, double‐blind, controlled study, AG10 was uniformly well tolerated with no safety concerns in healthy volunteers and was able to stabilize TTR across dosing intervals; these findings suggest AG10 may be a safe and effective treatment for either mutant or wild‐type ATTR.^51^ In another pharmacological study, AG10 had more hydrogen bonding interactions than did tafamidis, indicating a stronger bonding interaction with the tetramer receptors and possibly more TTR stability compared to tafamidis.^52^

#### 6.1.3 Drugs that inhibit oligomer aggregation and tetramer dissociation

##### Epigallocatechin gallate (EGCG)

EGCG is a catechin in green tea that was found to bind to soluble TTR and decrease the frequency of tetramer dissociation in vitro.^53^ In an observational study in 19 patients conducted by Kristen et al,^54^ EGCG halted progression of cardiac TTR amyloidosis. In a phase 2 trial, daily oral EGCG was used to treat AL amyloidosis, with favorable clinical efficacy and low toxicity.^55^ However, results from a single‐center retrospective study suggested that EGCG, although a safe therapeutic option, was not associated with improved survival.^56^ No clinical trial of EGCG as a treatment for ATTR has been conducted to date.^57^

#### 6.1.4 Drugs affecting degradation and reabsorption of amyloid fibers

##### Doxycycline‐tauroursodeoxycholic acid (Doxy/TUDCA)

Doxy/TUDCA appears to be effective in vitro in causing complete disaggregation of amyloid fibers and generating nontoxic molecular species.^58^ In a small phase 2 open label study, treatment with Doxy/TUDCA was well tolerated and resulted in no progression of cardiac involvement and neuropathy.^58^ The preliminary data showed that Doxy/TUDCA stabilizes the disease for 1 year with an acceptable toxicity profile.^58^ Doxy/TUDCA is now being tested in a phase 3 clinical trial (NCT03481972).^58^, ^59^

##### Miridesap

Miridesap is an antibody that targets serum amyloid P protein (SAP) or amyloid fibrils. SAP, a normal plasma glycoprotein synthesized by the liver, stabilizes and protects amyloid fibrils from proteolytic degradation. Miridesap binds SAP and promotes its hepatic clearance.^43^ A clinical trial of miridesap showed improved cardiac function with no significant cardiac side effects reported, but the trial was stopped on 22 August 2018, by the pharmaceutical company due to a change in benefit/risk profile (CT03044353).^43^, ^60^

##### PRX004

PRX004 is a monoclonal antibody designed to specifically target TTR amyloid deposits and is currently being tested in a phase 1 trial.^61^ These antibodies inhibit TTR fibrillogenesis and induce antibody‐dependent phagocytic uptake of TTR aggregates in vitro.^62^ Future study is warranted to assess its efficacy in animal models.

###### Dezamizumab

Dezamizumab is a humanized monoclonal IgG1 anti‐SAP antibody that is being studied for treating amyloidosis in a phase II trial.^63^ It binds to SAP in amyloid deposits and activates complement, thus triggering the clearance of the amyloid.^63^ The trial for dezamizumab was terminated due to a change in benefit/risk profile.^64^

### 6.2 Other treatments for advanced‐stage ATTR

#### 6.2.1 Liver transplantation and combined liver‐heart transplantation

Once a diagnosis of ATTR is confirmed, the patient should be evaluated for liver transplantation, as the source of amyloidogenic protein is the liver. However, Dubrey and colleagues found that nearly half of patients with family amyloidosis still developed evidence of amyloid cardiomyopathy after liver transplantation.^65^, ^66^ In a single‐center study at the Mayo Clinic, patients with combined heart and liver transplantation demonstrated excellent outcomes with 10‐year survival rates of 60%.^65^ Given the low number of transplants, however, data is limited in terms of overall outcomes among patients who have received combined heart and liver transplantation.

### 6.3 Treatment of comorbidities and complications

#### 6.3.1 Heart failure

##### Medical treatment

Traditional drug therapy for congestive heart failure is generally used for patients with ATTR, although there is no evidence that it affects the overall prognosis of amyloidosis. Patients with ATTR typically respond better to heart failure therapy than do those with AL amyloidosis.^20^ Medications often used are diuretics, beta blockers, and angiotensin‐converting enzyme inhibitors or angiotensin receptor blockers. Notably, nondihydropyridine calcium channel blockers are contraindicated due to their negative inotropic effect. Digoxin is not often used in practice due to its toxic profile (Table 2).

**TABLE 2:** Treatment for transthyretin‐related amyloidosis and its comorbidities and complications

Medical treatment for ATTR	Invasive treatment for end‐stage ATTR	Treatment for comorbidities and complications
Inhibitors of TTR gene expression Tetramer stabilizers Inhibitors of oligomer aggregation and disruption Inhibitors of degradation and reabsorption of amyloid fibers	Left ventricular assist device and heart transplant (only if no extracardiac involvement) Liver transplantation Combined liver‐heart transplantation	1. Heart failure a) Medical treatment Diuretics Beta blockers Angiotensin‐converting enzyme inhibitors/angiotensin receptor blockers (Nondihydropyridine calcium channel blockers are contraindicated due to their negative inotropic effect.) b) Left ventricular assist device: bridging for heart transplant 2. Atrial fibrillation Role of anticoagulation is unclear Cardioversion remains a reasonable treatment option 3. Life‐threatening arrhythmia a) ICD Role of ICD as primary prevention of sudden cardiac death unclear given lack of overall survival benefit b) Cardiac pacing Indication of PPM placement is the same as current guideline Limited evidence of survival benefit

Abbreviations: ATTR, transthyretin‐related amyloidosis; ICD, implantable cardioverter‐defibrillator; PPM, permanent pacemaker; TTR, transthyretin.

##### Left ventricular assist device and cardiac transplantation (end‐stage amyloidosis treatment)

Left ventricular assist device and cardiac transplantation are options for patients with ATTR cardiomyopathy‐related end‐stage heart failure with no significant extracardiac involvement, based on United Network for Organ Sharing (UNOS) status. However, evidence of survival benefit is lacking given the limited number of cardiac transplants performed due to the scarcity of donor organs. Davis et al at Stanford University Hospital conducted a study in 19 patients undergoing heart transplantation for amyloid cardiomyopathy between 2008 and 2013.^67^ After a median follow‐up of 380 days, the overall survival rate was 89.5% (74%, ATTR patients; 100%, AL patients). According to the UNOS registry, the 1‐year survival of patients undergoing heart transplant for amyloidosis was 74.5% compared with 81.5% for those undergoing heart transplant for other etiologies of restrictive cardiomyopathy, indicating an inferior survival rate in amyloidosis cardiomyopathy.^68^ Limitations of this study include its small sample size and the combined assessment of patients with ATTR amyloidosis and AL amyloidosis.^68^

##### Atrial fibrillation and intracardiac thrombosis

Some patients with cardiac amyloidosis have received anticoagulats because of the increased risk of intracardiac thrombosis, especially those with AL amyloidosis.^69^ However, it is unclear if anticoagulation leads to improved outcomes in ATTR patients.^69^ Cardioversion is a reasonable choice for atrial fibrillation and rhythm control, but maintaining sinus rhythm is challenging.^70^

##### Life‐threatening arrhythmia

###### Implantable cardioverter‐defibrillator

Previous studies have demonstrated that life‐threatening arrhythmia‐related sudden cardiac death (SCD) is a common cause of death in patients with cardiac amyloidosis.^71^ Currently, there is no evidence suggesting that an implantable cardioverter‐defibrillator (ICD) improves survival.^72^ It is suspected that ICD therapy may not prolong survival in patients with amyloid cardiomyopathy because most SCDs are caused by electromechanical dissociation rather than a potentially reversible ventricular arrhythmia.^72^, ^73^ In addition, as consensus guidelines recommend against ICD placement for the primary prevention of SCD in patients with a life‐expectancy of less than 1 year, ICDs have typically not been implanted in ATTR cardiomyopathy patients with arrhythmia or heart failure, whose median survival is often less than 1 year.^74^

##### Cardiac pacing

The infiltration of myocardial tissue into the conduction system often leads to lethal arrhythmias. Electrocardiogram abnormalities among patients with amyloidosis warrant an electrophysiological workup. Pacing is primarily used for significant bradycardia and certain types of atrial‐ventricular blocks, as a result of myocardial tissue infiltration. The data are limited on the indication for pacemaker placement and the clinical outcomes among this subgroup of patients.

## 7 PROGNOSIS

The prognosis of patients with ATTR is determined primarily by the presence and extent of cardiac involvement.^15^ In a study by Escher et al, the dominant cause of death in ATTR cardiomyopathy was cardiovascular failure, with 80% of cases featuring acute HFpEF.^75^ Regardless of the type of amyloidosis, the onset of advanced heart failure symptoms portends a poor prognosis; median survival ranges from 6 months in light chain amyloidosis to 43 months in ATTR‐wt.^76^, ^77^

In a retrospective study, Escher et al followed and assessed 5‐year mortality in 325 patients with confirmed cardiac amyloidosis.^75^ The 30‐month mortality rate was 65% for AL amyloidosis and 25% for the ATTR‐wt group. In their study, prognosis was likely confounded by how the patient was treated, as four patients (1.2%) underwent heart transplantation, at least 28.2% patients received anticoagulation due to atrial fibrillation, and 25% of patients were treated with an ICD and/or pacemaker.^75^

## 8 CONCLUSION

Once considered incurable, ATTR cardiomyopathy, a subtype of amyloidosis‐associated cardiomyopathy, now has promising treatment alternatives. Currently, robust ongoing research and clinical trials are focused on identifying new treatments for ATTR cardiomyopathy. Most of the novel agents target amyloid fiber formation by inhibiting TTR gene expression, stabilizing the TTR tetramer, preventing oligomer aggregation, or affecting degradation of amyloid fibers. More research is needed to verify the mortality benefits and side effects of the medications. It is unclear if the indication for heart failure treatment, anticoagulation, ICD placement, and transplant are the same as in those without ATTR. In addition, there is no consensus in screening criteria for ATTR, and the diagnosis of ATTR cardiomyopathy remains challenging because its nonspecific manifestations can be easily categorized as HFpEF. With the use of new treatments, this significantly underdiagnosed disease will likely be revealed as more prevalent than once thought. Therefore, further study is warranted to clarify the screening, diagnosis, and treatment guidelines for ATTR patients with cardiomyopathy.

## CONFLICT OF INTEREST

The authors have no actual or potential conflict of interest in relation to this manuscript.
